# Sleeping Sickness

**Published:** 1908-10-24

**Authors:** F. M. Sandwith


### Hospital Clinics.

### SLEEPING SICKNESS.

By F. M. SANDWITH, M.D., F.R.C.P.

Trypanosomiasis is a disease occurring in rats, horses, cattle, and many mammals, in birds, reptiles, frogs, toads, and fishes. When the disease attacks man (*human trypanosomiasis*) it is still usually called by the most noteworthy symptom of the terminal stage of the malady.

It is now exactly six years since Dr. Dutton showed to some of us the first case imported into this country. Since then it has been recognised by many clinical observers that the disease usually begins with irregular fever, skin eruptions, enlargement of cervical and other glands, while eventually physical and mental lethargy supervene. The earliest medical account of sleeping sickness dates from Sierra Leone in 1803, but it is believed that slave traders were well acquainted with the disease in yet earlier times. In this country the first case described was that of a negro at the London Hospital under the care of Sir Stephen Mackenzie, and in 1900 Dr. Mott pointed out that the lesion causing death was a chronic meningo-encephalitis.

The opening up of trade routes from West Africa to Uganda apparently led to a great epidemic in that Protectorate. Whole villages have been depopulated, and it is calculated that more than 200,000 of the inhabitants have died of this disease alone during the last six years.

It used to be considered that the malady was peculiar to coloured people, but this is very far from being the case. The French Commission to the Congo has found by microscopic examination of the blood that twenty Europeans are now infected, and more than a dozen Europeans have been treated in this country, besides others in France, Belgium, and Germany. No race or age is exempt from this terrible plague, but the disease is so far confined to those who have lived for some time in tropical Africa. Various Commissions have been despatched to Uganda, and in 1902 Dr. Castellani and Sir David Bruce succeeded in showing that the presence of trypanosomes in the peripheral blood is the cause of the disease.

The parasites are now proved to gain access to the human body by means of the tsetse fly (Glossina palpalis and Glossina fusca). They cause the irregular fever (at night to 103° or 104°, with morning remissions to the normal temperature). This is accompanied by a peculiar erythema and very often by a serous infiltration of the connective tissue of the erythematous patches.* The patients complain of headache, general debility, and tenderness of cervical and other glands, which are then found to be slightly enlarged. The glands vary in size from a small bean to a hazel nut, and can best be found in the anterior and posterior triangle of the neck, beneath the jaw, and in the inguinal regions. The trypanosomes can be found in juice withdrawn from the enlarged glands by a hypodermic syringe, and it has now become a rule to examine glands of palpable size in this way, before arriving at the diagnosis of sleeping sickness, in those parts of Africa where the disease is suspected. The early symptoms may continue for several months without giving rise to any cerebral manifestations, and it is probable that the latter only occur when the parasite has invaded the cerebro-spinal fluid.

The trypanosomes can then be found by removing about a quarter of an ounce of cerebro-spinal fluid by lumbar puncture, and examining the deposit carefully after centrifugalisation. Patients have a dull, apathetic look, and wait a considerable time before replying to any question. Excessive sleep is not such a predominant symptom as is generally supposed, for the usual condition is better described as one of lethargy; the patient, if left alone, is often seen to nod his head with eyes closed, but the slightest touch will cause him to open his eyes at once. The drowsiness gradually passes into coma, which deepens till the patients can no longer be roused, and in this state they die.

Some two years elapse from the earliest moment of discovering the parasite in the blood until death, but, in some acute cases in Uganda, patients have been known to die within six weeks of the first clinical symptoms.

The disease almost invariably terminates fatally, but one lady who received her infection during 1900 on the Congo, and in whose blood trypanosomes were discovered in the autumn of 1902, is to-day still alive and in excellent health. She was treated by arsenic, and there are other Europeans alive and apparently well after three years' treatment by atoxyl, which is a drug prepared from arsenate of aniline. The best routine treatment as yet known is atoxyl two or three times a week for at least two years. Patients so treated lose their fever and their rash, and the glands return to normal size; while trypanosomes can no longer be discovered in their blood or in the gland juice, and the injection of their blood into monkeys and rats does not cause trypanosomiasis in these animals.

The best methods of combating the disease are avoidance of the marshy places favoured by the tsetse fly; the destruction of the fly itself; the protection of healthy as well as infected persons from the sting of the fly; to prevent the introduction of diseased persons into healthy villages; and, lastly, to transfer the inhabitants of infected villages to districts where the fly does not exist. For the destruction of the fly it is necessary to cut down the brushwood for about five hundred yards from the water, and to deprive the insect of its usual food, that is, the blood of vertebrates.

In order to control the fight against the "fly disease" a sleeping-sickness bureau has been established in London, and a new Commission has already reached East Africa under the charge

* Visitors to the Science Section of the Franco-British Exhibition can see in the exhibit of the London School of Tropical Medicine a plaster cast and a water-colour sketch showing this eruption on a white skin.

of Sir David and Lady Bruce, accompanied by Captain Hamerton and Captain Bateman, R.A.M.C. The mission will proceed to the northern shore of Lake Victoria, where a laboratory is being prepared within five miles of one of the concentration camps organised by the Government in which sleeping sickness patients are under treatment.

The research work will include the study of the natural history of the fly and of Professor Koch's theory that crocodiles are its chief food. It is also hoped that some light may be thrown on the question as to whether the fly simply transfers the parasite from one animal to another or whether a cycle of development takes place within it analogous to that of the malaria parasite within the mosquito.

